# Treatment of Locally Advanced Prostate Cancer: A Case Report and Narrative Review

**DOI:** 10.1155/2012/402513

**Published:** 2012-12-17

**Authors:** Frank Peinemann, Michael Pinkawa

**Affiliations:** ^1^Institute of Health Economics and Clinical Epidemiology (IGKE), University Hospital of Cologne, 50924 Cologne, Germany; ^2^Department of Radiotherapy, University Hospital Aachen, 52074 Aachen, Germany

## Abstract

*Introduction*. Treatment of locally advanced prostate cancer is under discussion. Differences between clinical and pathological staging and risk factors such as positive surgical margins and seminal vesicle involvement challenge the individual treatment decisions. *Case Presentation*. Clinical tumor stage before treatment was assessed to be T2. After radical prostatectomy, pathological examination revealed the stage pT3b N0 M0 including positive surgical margin and seminal vesicle involvement. Early adjuvant androgen deprivation therapy and late adjuvant radiation therapy were added in response to the pathological risk factors. No evidence of disease was observed for 15 years after the treatment. The unexpected pathological findings were not explained by the physicians in charge. *Discussion*. A narrative review of the recent literature showed that multiple treatment modalities including adjuvant radiotherapy following radical prostatectomy are consistent with current recommendations. The multimodal approach has possibly cured a high-risk patient and may also work successfully in other patients. An alternative treatment option with better preservation of health-related quality of life might have also achieved a similar good overall survival.

## 1. Introduction

Prostate cancer will be diagnosed in 2012 in an estimated 241,740 men; 28,170 men will die, and the lifetime risk being diagnosed is 16.48% (1 in 6) in the USA [1]. Locally advanced prostate cancer can be defined by the categories T3a, T3b, T4, or by the category N1 associated with any T of the Tumor-Node-Metastasis (TNM) staging system [2] if combined with an absence of distant metastasis (M0). Patients are regarded at high risk if the prostate cancer is locally advanced or the Gleason score is 8 to 10 or a serum prostate-specific antigen is greater than 20 ng/mL [3].

The tumor type presented in this paper has extended through the prostate capsule into the seminal vesicles (T3b N0 M0) and is therefore categorized as a very high-risk locally advanced prostate cancer (T3b N0 M0, T4 N0 M0, or any T N1 M0). Radical prostatectomy (RP) can be a reasonable first step treating very high-risk locally advanced prostate cancer in selected patients [3]. Microscopic metastases may be present but not yet detectable and there is a considerable risk of incomplete tumor removal. Lymph node disease (N1) is associated with a high risk for systemic tumor progression and treatment failure. Therefore, multiple treatment modalities such as extended pelvic lymph node dissection, adjuvant radiotherapy (RT), and adjuvant androgen deprivation therapy (ADT) can be offered.

## 2. Case Presentation

In 1995, a 61-year-old male presented lower urinary tract symptoms secondary to benign prostate hyperplasia to an outpatient urologist. Diagnostic data were in agreement with a benign prostatic obstruction based on benign prostatic enlargement. First, the urologist prescribed extracts of the saw palmetto plant (*Serenoa repens*), classified as a dietary supplement. One year later, treatment was changed to tamsulosin, an *α*1a-selective alpha blocker. Yohimbine, an alpha-2 adrenoceptor blocker, was added to treat erectile dysfunction.

In 1997, at age 63, following a suspicious digital rectal exam of the prostate gland, the concentration of prostate-specific antigen in blood was measured for the first time. The level was 9.1 ug/L. A transrectal ultrasound examination identified a conspicuous region. A consecutive guided biopsy of the tissue of this region revealed an adenocarcinoma of the prostate gland with a Gleason score of 4.

A secondary level of care hospital in Germany offered a radical prostatectomy (RP) because of young age and insignificant comorbidities. The size of the removed prostate gland was approximately 150 mL (5.3 cm diameter). Histopathological evaluation revealed an adenocarcinoma that had broadly penetrated the margins of the prostate gland. The tumor mass had bilaterally invaded into the surrounding soft tissue, the seminal gland, and the seminal tract. Furthermore, the urethra was also involved with tumor infiltration. Urinary bladder was not involved. The pathologist identified a wide positive surgical margin in the resection material. Preoperatively, computed tomography did not identify affected pelvic lymph nodes and total body skeletal scintigraphy did not identify distant bone metastases. The pathological staging of T3b N0 M0 (T3c N0 M0 of the TNM modification at that time) was compatible with a high-risk locally advanced prostate cancer. The Gleason score was not determined. 

An early androgen deprivation therapy (ADT) was offered using the gonadotropin-releasing hormone (GnRH) analogon leuprorelin. Due to severe adverse events, ADT was discontinued after eight months and followed by radiation therapy (RT) by a nearby tertiary level of care hospital with a total dose of 66 Gy within the next two months after discontinuation of ADT. The patient is alive with no evidence of disease after a followup of 15 years. A PSA level below 0.1 ng/mL for 15 years is compatible with complete remission and absent prostate cancer in 2012.

The patient expressed several complaints. RP was regarded by the physicians as the best choice. Treatment alternatives were not discussed and a shared-decision making was not was not offered. The patient complained mainly about loss of sexual function and enuresis. He asked whether these adverse effects could have been prevented and why he was not informed about the extensive impact of adverse effects. The patient had substantial difficulties to endure the pronounced adverse effect of androgen deprivation therapy. Temporary adverse effects of the patient included a urinary bladder neck stricture treated by electrocauterization, mood depression, emotional distortion, hair loss, and painful defecation. Treatment-related long-term adverse effects of the patient included erectile dysfunction, urinary and rectal incontinence, and gynecomastia. Health-related quality of life is reduced substantially. It is not known whether the sacrifice of sexual integrity was necessary to save the life.

The senior clinician of the hospital misled the patient about the unexpected pathological stage after operation. Hospital representatives informed the patient that everything is alright and that the prostate gland with its cancer tissue inside has been removed. Neither the invasion across the capsule into other organs nor the positive surgical margin and its implication of residual prostate cancer tissue in the body were addressed. The head of the urology department stated that he did not want to worsen the patient's depressed mood. Weeks after discharge, the patient learned about the outcome from a copy of the medical report, which was sent to the general practicioner. 

## 3. Discussion

We searched PubMed on February 05, 2012 using these search terms “Prostatic Neoplasms” (MeSH) AND “Prostatectomy” (MeSH) AND “locally advanced” (tiab) and retrieved 330 results. We retrieved two systematic reviews and 18 trials in The Cochrane Library using the same search terms. We used the Clinical Queries of PubMed using these search terms locally advanced prostate cancer radical prostatectomy and retrieved 219 results applying the *therapy/broad* filter and retrieved 29 results applying the *systematic review* filter. We evaluated recently published reviews and studies on locally advanced prostate cancer treated with prostatectomy. We retrieved 420 results in PubMed, PubMed Clinical Queries, and The Cochrane Library after removing 178 duplicates. We screened title and abstract and selected 23 articles for full text evaluation. We added further references identified by the PubMed Related Articles tool. 

Locally advanced prostate cancer is characterized by extracapsular extension including microscopic bladder neck involvement (pT3a) or invading the seminal vesicles (pT3b) or invading other adjacent organs (T4). Positive lymph nodes may be present but distant metastases should be ruled out.

Detection of prostate cancer and differentiation from benign prostate hyperplasia (BPH) depend on histopathological assessment enabled by prostate biopsy [4]. An abnormal digital rectal examination or an elevated serum concentration of prostate-specific antigen (PSA) is sufficient to recommend a biopsy. A cut-off level of 4.0 ng/mL [5] is applied in general and a cut-off level of <2.5 ng/mL is often used for younger men [3]. Clinical T staging of prostate cancer such as extraprostatic extension (cT3) is based on findings from digital rectal examination and is possible with magnetic resonance imaging. PSA level [5], Gleason score [6], tumor grade, and clinical stage are predictive of the outcome [4].

It is not possible to certainly differentiate a fast from a slow growing tumor. DRE often underestimates the presence of tumor, transrectal ultrasound (TRUS) is not useful for detection of tumor, and PSA is produced by benign and malignant prostatic tissue. The higher the PSA gets the higher the risk for any type of prostate cancer is. For example, the risk of prostate cancer for patients with “normal” PSA levels is estimated at 6.6% for levels 0 to 0.5 ng/mL and climbs up to 26.9% for levels 3.1 to 4 ng/mL [7]. In other words, 1 in every 7 men with PSA <4 ng/mL has prostate cancer, 5 out of 10 men with PSA 4 to <10 ng/mL has prostate cancer, and one of every two men with PSA higher than 10 have prostate cancer [8]. PSA velocity may be observed by urologists and a rise of 0.35 ng/mL/year is currently used as a cutoff [8]. However, PSA itself was found to be highly predictive of local advanced prostate cancer [9] and the recommendation that men with high PSA velocity should be biopsied in the absence of other indications is questioned [10]. 

About 20% of T3 tumors were found to be overstaged, that is, the pathological stage pT2 was found in a patient with a supposed clinical stage cT3 [11]. Also, a considerable number of patients may be understaged meaning that the pathological stage pT3 was found in a patient with a supposed clinical stage cT2. In one series, only 54.6% of those with presumed localized disease (stage T1 to T2) actually had organ-confined prostate cancer (stage pT2) [12].

In the recent update of the European Association of Urology (EAU) Guidelines of Prostate Cancer RP is recommended as an optional treatment for selected patients with local advanced prostate cancer stage T3a and a multimodal approach including RP and adjuvant RT might be indicated [3]. RP is regarded as a standard treatment primarily for stage T1 to T2. A combination of RT and ADT is recommended for stage T3 to T4 with RT dose of at least 74 Gy and ADT duration of two years. Followup is largely based on PSA. Adjuvant ADT following RT may provide local control and improved disease-free survival [13, 14]. Adjuvant RT may have a favorable impact on the outcome after RP [15, 16]. The decision about the appropriate treatment is difficult and should consider the patients' preferences, the gravity of clinical and pathological findings, its impact on prognosis, and the frequency and severity of adverse events.

Biochemical recurrence will affect a considerable number of patients. It is reported by Xylinas et al., 2010, in 15% to 53% after primary curative therapy [11] and by Chang and Cookson, 2006, in 40% after RP of localized prostate cancer [17]. Steuber et al., 2006, reported a 5-year biochemical recurrence rate of 37% for pT3aN0 and 67% for pT3bN0 for patients without adjuvant ADT or RT [18]. 

Overall survival was reported about 77% after RP: 90.2% at 7-year stages T3 to T4 [19], 77.0% at 10-year stage unilateral cT3a [20], and 78.7% at 10-year stages pT3N0 to pT3N1 (RP with adjuvant ADT and RT) [21]. Cancer specific survival was reported about 91% after RP: 90.2% at 7-year stages T3 to T4 [19], 91.6% at 10-year stage unilateral cT3a [20], and 90.9% at 10 years stages pT3N0 to pT3N1 (RP with adjuvant ADT and RT) [21]. 

After RP, a positive surgical margin was reported in 18.5% to 70.4% of patients with clinical stages ranging from pT2a to pT4 [22–26]. The margin status was identified as a significant predictor of outcome by Hsu et al., 2007, [20] and Ploussard et al., 2011, [25] but not by Mearini et al., 2010, [24]. Patients with positive surgical margins after RP may experience five-year progression rates between 36% and 50% [27].

After RP, seminal vesicle involvement was reported in 8.5% to 32.1% of patients with clinical stages ranging from pT2a to pT4 [22, 25]. Seminal vesicle involvement was identified as a significant predictor of outcome by Cho et al., 2011, [28]. 

Xylinas et al., 2010, reported 4% urinary incontinence and 46% erectile dysfunction after RP [11]. ADT is associated with severe adverse events, such as hot flushes, gynecomastia, breast pain, decreased libido, impotence, and gastrointestinal and hematological toxicities [13, 14]. Stephenson et al., 2012, reported 3% to 5% genitourinary and gastrointestinal toxicity associated with postoperative radiation therapy for advanced prostate cancer [15].

The patient described in the case report was understaged in cT2 with a low risk in agreement with a PSA level below 10 ng/mL. RP was offered because patient had a life expectancy of more than 10 years. This first-line approach is consistent with the current recommendation to offer RP as a standard treatment for the appropriate stages. The pathologic examination established a pT3 stage with a very high risk due to positive surgical margin and seminal vesicle involvement. Adjuvant ADT and RT are consistent with the current recommendation to offer a multimodal therapy. A PSA of less than 0.1 ng/mL within 15 years means a long lasting recurrence-free survival. An alternative treatment might have also led to a tumor control with better preservation of health-related quality of life. Further studies are needed to evaluate long-term health-related quality of life after RP and adjuvant RT versus primary RT without RP, so that the best possible treatment is chosen for our patients. According to current guidelines, patients with high risk disease should be well selected before the indication for primary RP [3].

## References

[1] Siegel R, Naishadham D, Jemal A (2012). Cancer statistics. *A Cancer Journal for Clinicians*.

[2] World Health Organization (2012). Chapter 3. tumours of the prostate. *World Health Organization Classification of Tumours. Pathology and Genetics of Tumours of the Urinary System and Male Genital Organs*.

[3] Heidenreich A, Bolla M, Joniau S (2012). *Guidelines on Prostate Cancer*.

[4] Heidenreich A, Bellmunt J, Bolla M (2011). EAU guidelines on prostate cancer. Part 1: screening, diagnosis, and treatment of clinically localised disease. *European Urology*.

[5] Cooner WH, Mosley BR, Rutherford CL (1990). Prostate cancer detection in a clinical urological practice by ultrasonography, digital rectal examination and prostate specific antigen. *Journal of Urology*.

[6] Egevad L, Granfors T, Karlberg L, Bergh A, Stattin P (2002). Prognostic value of the Gleason score in prostate cancer. *British Journal of Urology International*.

[7] Thompson IM, Pauler DK, Goodman PJ (2004). revalence of prostate cancer among men with a prostate-specific antigen level < or = 4.0 ng per milliliter. *The New England Journal of Medicine*.

[8] NCCN (2011). *NCCN Guidelines for Patients: Prostate Cancer, Version 1*.

[9] Ulmert D, Cronin AM, Björk T (2008). Prostate-specific antigen at or before age 50 as a predictor of advanced prostate cancer diagnosed up to 25 years later: a case-control study. *BMC Medicine*.

[10] Vickers AJ, Savage CJ, Bianco FJ (2011). Surgery confounds biology: the predictive value of stage-, grade- and prostate-specific antigen for recurrence after radical prostatectomy as a function of surgeon experience. *International Journal of Cancer*.

[11] Xylinas E, Daché A, Rouprêt M (2010). Is radical prostatectomy a viable therapeutic option in clinically locally advanced (cT3) prostate cancer?. *British Journal of Urology International*.

[12] Berge V, Thompson T, Blackman D (2007). Use of additional treatment for prostate cancer after radical prostatectomy, radiation therapy, androgen deprivation, or watchful waiting. *Scandinavian Journal of Urology and Nephrology*.

[13] Shelley MD, Kumar S, Coles B, Wilt T, Staffurth J, Mason MD (2009). Adjuvant hormone therapy for localised and locally advanced prostate carcinoma: a systematic review and meta-analysis of randomised trials. *Cancer Treatment Reviews*.

[14] Kumar S, Shelley M, Harrison C, Coles B, Wilt TJ, Mason MD (2006). Neo-adjuvant and adjuvant hormone therapy for localised and locally advanced prostate cancer. *Cochrane Database of Systematic Reviews*.

[15] Stephenson AJ, Bolla M, Briganti A (2012). Postoperative radiation therapy for pathologically advanced prostate cancer after radical prostatectomy. *European Urology*.

[16] Wiegel T, Bottke D, Steiner U (2009). Phase III postoperative adjuvant radiotherapy after radical prostatectomy compared with radical prostatectomy alone in pT3 prostate cancer with postoperative undetectable prostate-specific antigen: ARO 96-02/AUO AP 09/95. *Journal of Clinical Oncology*.

[17] Chang SS, Cookson MS (2006). Impact of positive surgical margins after radical prostatectomy. *Urology*.

[18] Steuber T, Erbersdobler A, Graefen M, Haese A, Huland H, Karakiewicz PI (2006). Comparative assessment of the 1992 and 2002 pathologic T3 substages for the prediction of biochemical recurrence after radical prostatectomy. *Cancer*.

[19] Gontero P, Marchioro G, Pisani R (2007). Is radical prostatectomy feasible in all cases of locally advanced non-bone metastatic prostate cancer? Results of a single-institution study. *European Urology*.

[20] Hsu CY, Joniau S, Oyen R, Roskams T, van Poppel H (2007). Outcome of surgery for clinical unilateral T3a prostate cancer: a single-institution experience. *European Urology*.

[21] Miyake H, Sakai I, Harada KI, Hara I, Eto H (2004). Long-term results of adjuvant hormonal therapy plus radiotherapy following radical prostatectomy for patients with pT3N0 or pT3N1 prostate cancer. *International Journal of Urology*.

[22] Berglund RK, Jones JS, Ulchaker JC (2006). Radical prostatectomy as primary treatment modality for locally advanced prostate cancer: a prospective analysis. *Urology*.

[23] Hsu CY, Wildhagen MF, van Poppel H, Bangma CH (2010). Prognostic factors for and outcome of locally advanced prostate cancer after radical prostatectomy. *British Journal of Urology International*.

[24] Mearini L, Zucchi A, Costantini E, Bini V, Nunzi E, Porena M (2010). Outcomes of radical prostatectomy in clinically locally advanced N0M0 prostate cancer. *Urologia Internationalis*.

[25] Ploussard G, Agamy MA, Alenda O (2011). Impact of positive surgical margins on prostate-specific antigen failure after radical prostatectomy in adjuvant treatment-naive patients. *British Journal of Urology International*.

[26] Schelin S, Madsen M, Palmqvist E, Mäkelä E, Klintenberg C, Aus G (2009). Long-term follow-up after triple treatment of prostate cancer stage pT3. *Scandinavian Journal of Urology and Nephrology*.

[27] Kamat AM, Babaian K, Cheung MR (2003). Identification of factors predicting response to adjuvant radiation therapy in patients with positive margins after radical prostatectomy. *Journal of Urology*.

[28] Cho IC, Kwon WA, Kim JE (2011). Prostate volume has prognostic value only in pathologic T2 radical prostatectomy specimens. *Journal of Korean Medical Science*.

